# Baseline factors that are associated with change in visual acuity in intermediate AMD over two years in a multicentre cohort study in Europe- INTERCEPT-AMD Report 2

**DOI:** 10.1038/s41433-025-04062-z

**Published:** 2025-10-17

**Authors:** Sarega Gurudas, Inês Marques, Jean-François Girmens, Yara Lechanteur, Mariacristina Parravano, Lieselotte Berger, Hansjürgen Agostini, Sandra Barrão, Evangelos Tsiroukis, Jordi Monés, Laura Sararols, Rufino Silva, Hendrik P. N. Scholl, Albrecht Lommatzsch, Boris Stanzel, Stela Vujosevic, Sobha Sivaprasad, the INTERCEPT-AMD Study Group, Yannick Liermann, Paolo Lanzetta, Emily Fletcher, Savita Madhusudhan, Lyubomyr Lytvynchuk, Francesco Bandello, Nicole Eter, Stefano de Cilla, Michel Weber, Aude Ambresin

**Affiliations:** 1 NIHR Moorfields Clinical Research Facility, Moorfields Eye Hospital, NHS Foundation Trust, London, UK; 2 Centre for Clinical Trials. AIBILI/Association for Innovation and Biomedical Research on Light and Image, Coimbra, Portugal; 3 Centre d’Investigation Clinique, Centre National d’Ophtalmologie des Quinze-Vingts, Paris, France; 4 Department of Ophthalmology Radboud University Medical Centre, Nijmegen, Netherlands; 5 IRCCS Fondazione G.B. Bietti per lo Studio e la Ricerca in Oftalmologia ONLUS, Rome, Italy; 6 Department of Ophthalmology, Inselspital, University of Bern, Bern, Switzerland; 7 Department of Ophthalmology, University of Freiburg, Freiburg, Germany; 8 Instituto de Oftalmologia Dr. Gama Pinto, Lisboa, Portugal; 9 Institut Català de Retina (ICR), Clinical Trial Unit, Barcelona, Spain; 10 Institut de la Màcula, Centro Médico Teknon, Barcelona, Spain; 11 Valles Ophthalmology Research, S.L, Barcelona, Spain; 12 Espaço Médico de Coimbra, Coimbra, Portugal; 13 University Hospital Basel, University Eye Clinic, Basel, Switzerland; 14 Department of Ophthalmology, St. Franziskus-Hospital, Münster, Germany; 15 Eye Clinic Sulzbach, Knappschaft Hospital Saar, Sulzbach, Germany; 16 Medical Retina Service, Operative Unit Ophthalmology - MultiMedica Spa (IRCCSMM), Milan, Italy; 17 Department of Ophthalmology University of Bonn, Bonn, Germany; 18 Department of Ophthalmology, University of Udine, Udine, Italy; 19 Clinical Trial Unit, Dep. Ophth., Gloucestershire Hospitals NHS Foundation Trust, Cheltenham, UK; 20 Clinical Eye Research Centre – St. Paul’s Eye Unit, Royal Liverpool University Hospital, Liverpool, UK; 21 Department of Ophthalmology, Justus-Liebig-University-Giessen, Giessen, Germany; 22 Department of Ophthalmology, University Vita Salute - Scientific Institute of San Raffaele, Milan, Italy; 23 Department of Ophthalmology, University of Muenster Medical Center, Münster, Germany; 24 Eye Unit, University Hospital Maggiore della Carità, Novara, Italy; 25 Department of Ophthalmology, University Hospital, Nantes, France; 26 Swiss Visio Retina Research Center, Swiss Visio Montchoisi, Lausanne, Switzerland

**Keywords:** Biomarkers, Predictive markers

## Abstract

**Background/Objectives:**

The aim of this study was to evaluate the change in visual acuity (VA) in participants with intermediate age-related macular degeneration (iAMD) over two years when categorised by the presence or absence of incomplete retinal and retinal pigment atrophy (iRORA) and subretinal drusenoid deposits (SDD).

**Subjects/Methods:**

In this multicentre cohort study, participants with iAMD were classified as: i) iAMD with no iRORA or SDD; ii) iAMD with SDD with no iRORA; iii) iAMD with iRORA with no SDD and iv) iAMD with iRORA and SDD. The change in best recorded visual acuity (BRVA) over two years in the whole cohort and in each sub-category was analysed using linear mixed effect models employing an unstructured covariance structure. Associations with age, sex and baseline BRVA were evaluated.

**Results:**

983 eyes from 805 participants were analysed. The mean baseline VA changed from 79.8 (SD 8.1) to 77.7 (SD 10.1) Early Treatment Diabetic Retinopathy Study (ETDRS) letter score at two years, with a mean unadjusted change in BRVA of −0.33 ETDRS letters (95% CI −0.72,0.06); *P* = 0.10 at 6 months, −0.65 ETDRS letters (95% CI −1.14, −0.16); *P* = 0.01 at 12 months, −1.45 ETDRS letters (95% CI −2.03, −0.86); *P* < .001 at 18 months and −2.16 ETDRS letters (95% CI −2.79, −1.53); *P* < .001 at 24 months. All sub-categories showed a small decline in BRVA. Increasing age and lower baseline visual acuity were associated with lower BRVA over two years.

**Conclusion:**

Eyes with iAMD experience a small mean decline of ~2 letters over two years. Overall, these changes may not represent a clinically meaningful difference.

## Introduction

Age-related macular degeneration (AMD) is a degenerative retinal disease that is predicted to affect 288 million elderly people worldwide by 2040 [1]. The advanced forms are geographic atrophy (GA) and neovascular AMD (nAMD). Despite available treatments for both these conditions, they are leading causes of severe central visual impairment in the elderly [2]. Prevention of progression to advanced AMD is key to preventing visual loss. And several clinical trials are on-going in this area.

The presence of intermediate AMD (iAMD) is an established ocular risk factor for disease progression to advanced stages. Based on colour fundus photography of the macula, iAMD is characterised by at least one large druse (≥125 μm) and/or macular retinal pigment epithelial (RPE) disturbances associated with at least medium drusen [3]. However, most eyes with iAMD present with good visual acuity (VA) and decline in VA over time is slow. In order to design efficient clinical trials in iAMD, the change in VA over a short time frame in the control arm needs to be established, ideally in a natural history study.

Despite the slow natural history of vision loss in iAMD, prevention of 3-line loss of VA or 15 Early Treatment Diabetic Retinopathy Study (ETDRS) loss is the acceptable regulatory endpoint for clinical trials on ocular therapies in this area [4]. Another important point is that a minimum clinical important difference in VA is a change of at least 10 letters and for non-inferiority trials, an inferiority margin of about 5 letters is acceptable [4–6]. However, all these endpoints are challenging to achieve in clinical trials in iAMD because of good VA at baseline. A statistically significant difference between treatment and control arm in a superiority trial may be an option. An enriched study cohort with those at risk of VA loss in a short period of 1–2 years may be required.

Several risk factors for fast progressors in eyes with iAMD have been identified on optical coherence tomography (OCT) but their associations with change in VA in a short period is unclear. These OCT features include drusen size, morphology, and volume; hyperreflective foci; subretinal drusenoid deposits (SDD), nascent GA, and incomplete retinal pigment epithelium and outer retinal atrophy (iRORA) [7, 8]. As these factors may co-exist, the presence of a combination of these changes may help identify a sub-group at risk of faster VA decline. Therefore, categorising iAMD into various subgroups including more than one structural precursor of disease progression may improve our understanding on VA outcome in iAMD. As age of an individual and the disease state of the fellow eye are established risk factors for disease progression, these factors must also be considered in any risk models assessing VA loss in iAMD.

INTERCEPT AMD is a collaborative network study conducted by European Vision Institute for Clinical Research (EVICR.net) Members in Europe that were willing to develop a multimodal image database of eyes with iAMD in at least one eye with at least three sets of available images over a two-year follow-up in a user-friendly EVICR.net Eye Platform. The aim of this study was to assess VA change in iAMD over two years by phenotyping iAMD into four categories using multimodal images. The associations of baseline age, gender, VA, fellow eye status, iAMD phenotype in study eye with VA change at 2 years were evaluated.

## Methods

### Design

The study was managed by EVICR.net, Coimbra, Portugal that obtained the overarching institutional review board approval for the study. The EVICR.net also established data sharing agreements from sites to the EVICR.net Eye Platform and data and image transfer to Moorfields Eye Hospital via the EVICR.net Eye Platform. Each site then obtained their own regulatory approval required for each country, where applicable. The study protocol was approved by AIBILI Ethics Committee for approval. Informed consent from participants was obligatory only in some sites based on their regulatory approval while other countries considered this study as retrospective collection of routinely collected data and were exempt from further regulatory approval and informed consent.

### Selection of participants

Each participant with iAMD was identified by site investigators from respective electronic medical records or imaging datasets. To be eligible for this study, at least one eye of the participant had to have a diagnosis of iAMD with records of at least 3 clinic visits with multimodal imaging across two years. Multimodal imaging included mandated Spectralis macular OCT scans with or without enhanced depth imaging and infrared imaging. Fundus autofluorescence was optional. Eyes with poor-quality images were excluded. Other exclusion criteria included eyes with bilateral nAMD or geographic atrophy, any other co-existent ocular diseases including other types of macular atrophy or any individual who had opted out of their information being used for research nationally or locally at any Member Site (Supplementary Table S5). These criteria were screened by site personnel and reasons for exclusion as a study eye were recorded, where possible.

### Selection of study eye

If only one eye had iAMD, the macula status of the non-study eye was graded on multimodal imaging as presence of complete retinal and retinal pigment epithelial atrophy (cRORA), nAMD, early AMD, healthy macula or ungradable due to poor image quality or have other macular pathology. If both eyes meet the eligibility criteria of iAMD, they were both enrolled as study eyes.

In order to subclassify iAMD, the Classification of Atrophy Meetings group was used to define iRORA and cRORA on OCT [9]. SDD were identified in at least two imaging modalities [10]. The presence of SDD was defined as having ≥5 definite SDD on more than one B-scan within the central 20° × 20° region on OCT imaging and confirmed on infrared reflectance. Each eye with iAMD was further classified by the site personnel into the following pre-defined four categories at all three visits: i) Eyes with iAMD with no evidence of iRORA or SDD (considered as the eye with slowest rate of progression), ii) iAMD with SDD and no iRORA; iii) iAMD with iRORA and no SDD and iv) iAMD with iRORA and SDD. In addition, age and AMD severity level of the fellow eye were collected as they are strong predictors of disease progression.

### Change in VA by disease state at two years

The VA was recorded as ETDRS letter score at all sites. Methods of measurement of VA was recorded from the medical records from the index date and included VA with pinhole, VA with participant’s glasses or best-corrected visual acuity (BCVA) if participants were refracted at the time of their VA assessment. For the purposes of this study, this is collectively referred to as best recorded visual Acuity (BRVA). The eye with better and worse BRVA was defined as the better seeing eye or worse seeing eye if a participant-level BRVA was considered. The BRVA change was analysed for the whole cohort as well as based on key demographic features, baseline study eye and fellow eye disease status. The change in BRVA over time in the better and worse seeing eye at baseline were analysed.

### Sample size

A formal sample size estimation based on outcomes was not done as this study was aimed to collect a large research database for future studies. A sample size of 1000 eyes with BRVA records at baseline and over 2 years was considered a feasible resource for studying progression of different categories of iAMD and change in BRVA.

### Statistical analysis

Summary statistics for VA across 2 years (baseline, months 6, 12, 18 and 24), stratified by baseline BRVA, age, sex, study eye diagnosis, and fellow eye diagnosis, were presented as mean (SD). Participant and eye-level characteristics for the total cohort of eyes were summarised using mean (SD) or median (IQR) for continuous variables and *n* (%) for categorical variables.

The regression to the mean effect between baseline and month 24 VA was calculated as the percentage of regression to the mean (PRM) using the formula: 100× (1-*r*), where *r* is the pearson correlation coefficient between the baseline and month 24 measurements. The Linear mixed effects model (LMEM) for the continuous outcome BRVA score employing an unstructured covariance structure to account for the within-participant correlation between repeated measures over time (with time handled as a categorical variable) and the nesting between eyes from the same participant. Differences in BRVA, 95% confidence intervals with p-values were summarised from the models. Fixed effects that were adjusted for in LMEM’s for BRVA include the main effects of study eye diagnosis stage, fellow-eye diagnosis (nAMD or GA), age and sex and their interaction with time (defined as discrete visits at baseline, 6, 12, 18 and 24 months, i.e. as a categorical variable) in separate LMEM’s. Both unadjusted and age-sex adjusted analyses were presented. Baseline BRVA was not adjusted for, when reporting mean difference for the other variables but instead were modelled as part of the outcome, consistent with growth curve modelling approaches. Furthermore, baseline BRVA is considered a mediator for the relationship between demographic factors, study eye diagnosis and BRVA over time, therefore analysis of covariance could lead to biased estimates in mean difference in BRVA [11]. A LMEM assessing the association between baseline BRVA and BRVA at follow-up visits (6, 12, 18 and 24 months, i.e. excluding baseline from the outcome) was also modelled to demonstrate the strength of baseline BRVA as a predictor for future BRVA. Models were accompanied with line plots that show the trajectory of BRVA across time, stratified by study eye diagnosis. The proportion of 10-letter and 15-letter losers at 2 years was summarised. Associations with 10-letter and 15-letter losers were analysed using unadjusted and age-gender adjusted logistic regression models via generalised estimating equations (GEE), with Odds Ratios (ORs), 95% CI and *p*-values reported from the models.

For the analysis of eyes with unilateral eligibility (nAMD or GA in the fellow eye), within-participant correlations were based solely on repeated measures over time, as there is no nesting of data between the eyes. For the logistic regression models for variables exudative nAMD or GA in the fellow eye, these were fitted using generalised linear models rather than generalised estimating equations.

In sensitivity analysis, the natural history of BCVA, both overall and stratified by study eye diagnosis, was examined as the outcome instead of BRVA. This analysis was conducted on the subset of participants with available BCVA measurements. In a second sensitivity analysis, the natural history of BRVA, both overall and by study eye diagnosis, was evaluated after setting BRVA in study eyes that developed nAMD or GA during the study period to missing at the follow-up time in which the eye converted to nAMD or GA. This approach was used to assess the sensitivity of the results to potential declines in BRVA associated with these conditions. Baseline age and BRVA were analysed as categorical and continuous variables. The following groupings were followed for age; <75, 75–84, ≥85 years and baseline BRVA; ≤69 [worse than 20/40], 70–79 [20/40 to worse than 20/25], and 80 or better [20/25 or better] ETDRS letters [approximate Snellen].

All statistical analyses were performed using R, with a significance level set at 5% for all tests. Complete case analysis was carried out.

## Results

A total of 819 participants were evaluated. Of these, 983 eyes of 805 participants met the eligibility criteria for iAMD, excluding 17 (1.7%) eyes classified as other macular atrophy less than 250 microns typically those due to vitelliform maculopathy (Supplementary Table S1). The mean age was 75.8 years (SD 7.9 years), with 282 (35.0%) males. Mean age was highest in eyes with SDD (78.0, SD 7.6 in iRORA with SDD and 77.1, SD 75 in no atrophy with SDD) compared to without (74.0, SD 7.8 in no atrophy & no SDD and 72.8, SD 8.5 in iRORA & no SDD). Mean baseline BRVA was similar across iAMD phenotypes. BRVA was measured in a total of 950 (96.6%), 645 (65.6%), 861 (87.6%), 609 (62.0%) and 961 (97.8%) participants at baseline, 6 months, 12 months, 18 months and 24 months respectively. Visit windows were relatively narrow and consistent across time points (~±3 months for each visit): –3.22 to +3.33 months at 6 months, –3.60 to +3.60 months at 12 months, –2.83 to +3.40 months at 18 months, and –3.53 to +4.17 months at 24 months.

The mean baseline BRVA of the cohort was 79.8 (SD 8.1) [median VA 80.0 (IQR 75.0–85.0)] ETDRS letter score; with 630 (66.3%) eyes having BRVA 80 ETDRS letter score or better, while 3 (0.3%) had BRVA < 37 ETDRS letter score. Baseline BRVA was similar across the four categories of iAMD (Table 1).

**Table 1:** Mean best recorded visual acuity (BRVA) across time overall and by study eye diagnosis.

Variable	Baseline		M6		M12		M18		M24	
	*N*	Mean (SD)	*N*	Mean (SD)	*N*	Mean (SD)	*N*	Mean (SD)	*N*	Mean (SD)
Overall	950	79.8 (8.1)	645	79.4 (7.9)	861	79.2 (8.4)	609	78.2 (9.2)	961	77.7 (10.1)
Baseline BRVA, ETDRS letters [approximate Snellen]	–	–								
*≤69 [worse than 20/40]*			44	63.9 (11.3)	59	67.9 (12.1)	45	65.7 (12.8)	67	66.9 (12.7)
*70–79 [20/40 to 20/25)*			163	75.4 (6.6)	222	74.3 (8.1)	155	73.7 (9.7)	249	72.6 (10.6)
*80 or better [20/25 or better]*			424	82.6 (4.6)	561	82.5 (5.4)	391	81.4 (6.2)	619	81.0 (7.6)
iAMD diagnosis										
No iRORA & no SDD	315	80.3 (7.8)	198	80.0 (6.4)	286	79.8 (7.9)	179	78.3 (9.6)	315	78.2 (10.1)
No iRORA & SDD	367	79.5 (8.5)	268	79.5 (8.2)	341	79.4 (7.8)	261	78.7 (7.8)	374	77.3 (9.2)
iRORA & no SDD	110	80.5 (7.4)	83	80.0 (7)	96	80.2 (7.1)	73	80.0 (9.8)	114	79.2 (10.3)
iRORA & SDD	158	78.9 (8.2)	96	77.1 (10)	138	76.9 (11)	96	75.5 (10.7)	158	76.3 (11.8)
nAMD in fellow eye in unilateral iAMD^a^										
Absent	124	79.6 (9.8)	69	80.2 (7.8)	106	79.7 (8.4)	61	77.2 (11.8)	123	77.2 (13.2)
Present	490	78.8 (8.4)	367	79.1 (8.4)	461	78.0 (9.0)	344	77.8 (9.8)	490	76.8 (10.4)
GA in fellow eye in unilateral iAMD^a^										
Absent	544	78.7 (8.8)	394	78.9 (8.5)	505	78.0 (9.1)	369	77.3 (10.4)	543	76.5 (11.3)
Present	70	80.9 (7.2)	42	82.4 (4.4)	62	81.4 (5.8)	36	81.4 (5.5)	70	80.2 (7.9)
Age, years										
<75	417	82.0 (7.6)	271	81.5 (7.1)	379	81.3 (7.1)	259	80.7 (7.2)	429	80.0 (8.8)
75-84	427	78.6 (7.4)	297	78.6 (7.2)	385	78.2 (8.1)	277	76.8 (9.4)	424	76.1 (10.4)
85+	106	75.1 (9.8)	77	75.1 (10.6)	97	75.1 (11.5)	73	74.5 (11.9)	108	74.3 (12.1)
Sex										
Female	626	79.9 (7.7)	424	79.6 (7.8)	567	79.4 (8.7)	400	78.3 (9.3)	631	77.8 (10.3)
Male	324	79.5 (8.8)	221	79.0 (8)	294	78.9 (7.8)	209	78.0 (9.1)	330	77.3 (9.8)

*iAMD* intermediate age-related macular degeneration, *iRORA* incomplete retinal and retinal pigment epithelial atrophy, *SDD* subretinal drusenoid deposits, *GA* Geographic atrophy, *ETDRS* Early treatment Diabetic Retinopathy Study, *BRVA* best recorded visual acuity.

^a^nAMD and GA in the fellow eye were summarised in *N* = 627 eyes with unilateral iAMD. In these unilateral eyes, those with available data in BRVA were *N* = 614 at baseline, 436 at M6, 567 at M12, 405 at M18, and 613 at M24.

### Change in BRVA across 2 years

**Fig. 1:**
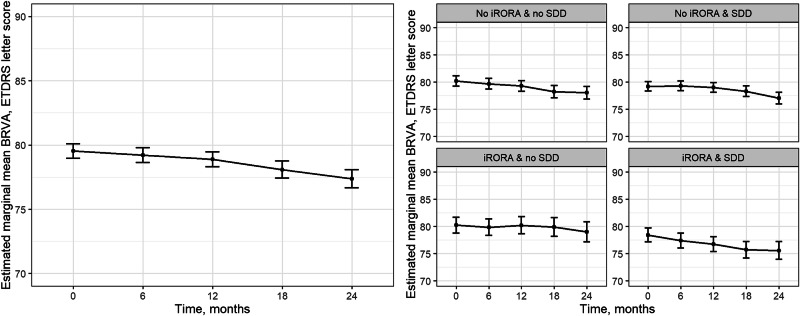
Longitudinal trajectory (natural history) of best recorded visual acuity (BRVA) overall and by iAMD diagnosis using linear mixed-effects models (LMEM’s)^a^. iAMD intermediate age-related macular degeneration, iRORA incomplete retinal and retinal pigment epithelial atrophy, SDD subretinal drusenoid deposits, VA visual acuity, ETDRS Early treatment Diabetic Retinopathy Study, BCVA best corrected visual acuity, BRVA visual acuity, LMEM Linear mixed-effects models. ^a^Number of eyes contributing BRVA data at each time point in the LMEM: Baseline (*N* = 950), M6 (*N* = 645), M12 (*N* = 861), M18 (*N* = 609) and M24 (*N* = 961). Error bars represent lower and upper limits of 95% asymptotic confidence intervals.

The Pearson correlation coefficient between baseline and 24-month BRVA was *r* = 0.54, corresponding to a 46.5% regression to the mean effect. This indicates that approximately 46.5% of the observed change may be attributable to regression to the mean. The mean BRVA at month 24 was 78 (SD 10.1) ETDRS letter score. Predicted BRVA in unadjusted LMEM show a decline in BRVA in the total cohort, and by study eye diagnosis across 2 years (Fig. 1). The mean unadjusted change in BRVA was −0.33 ETDRS letters (95% CI −0.72, 0.06); *P* = 0.10 at 6 months, −0.65 ETDRS letters (95% CI −1.14, −0.16); *P* = 0.01 at 12 months, −1.45 ETDRS letters (95% CI −2.03, −0.86); *P* < .001 at 18 months and −2.16 ETDRS letters (95% CI −2.79, −1.53); *P* < 0.001 at 24 months.

### BRVA based on better and worse seeing study eye

Table 2 demonstrates the mean baseline BRVA, mean BRVA at year 2 and change in BRVA based on better or worse seeing eyes. By 2 years, in eyes with bilateral iAMD, the worse seeing eye changed by −2.8 ETDRS letters (SD 5.1) compared to −1.2 ETDRS letters (SD 7.1) in the better seeing eye (*P* = 0.003). At month 24, study eye BRVA was 76.8 ETDRS letter score (SD 10.4) in eyes with nAMD in the fellow eye compared to 80.2 ETDRS letter score (SD 7.9) in study eyes with GA in the fellow eye (*P* = 0.002). Study eyes with nAMD in the fellow eye changed by −2.1 ETDRS letters (SD 9.6) compared to −0.7 ETDRS letters (SD 8.8) in study eyes with GA in the non-study eye (*P* = 0.23).

**Table 2:** Best recorded visual acuity (BRVA) based on disease state in study eye with iAMD at baseline.

		Baseline			2 years			Change		
		*N*	Baseline BRVA, mean (SD)	*P*-value	*N*	BRVA at 2 years, mean (SD)	*P*-value	*N*	Change in BRVA at 2 years, mean (SD)	*P*-value
Bilateral eyes	BRVA in worse seeing eye in bilateral AMD (study eye)	178	81.3 (6.8)	<0.001^a^	166	78.0 (8.8)	<0.001^a^	166	−1.2 (7.1)	0.003^a^
	BRVA in better seeing eye in bilateral AMD (study eye)		83.5 (5.5)			80.6 (6.2)			−2.8 (5.1)	
Unilateral eyes	BRVA in iAMD study eye (with nAMD in non-study eye)	490^c^	78.8 (8.4)	0.02^b^	490^c^	76.8 (10.4)	0.002^b^	484^c^	−2.1 (9.6)	0.23^b^
	BRVA in iAMD study eye (with GA in non-study eye)	70^c^	80.9 (7.2)		70^c^	80.2 (7.9)		68^c^	−0.7 (8.8)	

*iAMD* intermediate age-related macular degeneration, *nAMD* neovascular age related macular degeneration, *GA* geographic atrophy, *BRVA* Best recorded visual acuity.

^a^Paired *t*-test for BRVA comparing worse seeing and better seeing eye in bilateral AMD.

^b^Unpaired *t*-test for unilateral iAMD.

^c^ BRVA change data are missing in 6 nAMD and 2 GA non-study eyes due to differing patient sets at baseline and year 2 even though there are *n* = 490 and *n* = 70 in nAMD and GA at both baseline and 2 years.

### Associations for BRVA over time by baseline iAMD diagnosis, participant demographic variables and fellow eye status

Increased age, lower baseline BRVA and the absence of GA in the fellow eye were associated with reduced BRVA at all follow-up time points (Table 3 and Supplementary Fig. S2). In age-sex adjusted models, at 2 years, every 1-year increase in baseline age was associated with a −0.34 ETDRS letter difference (95% CI −0.42, −0.25; *P* < 0.001) in BRVA (Table 3). Eyes with baseline BRVA < = 69 ETDRS letters relative to eyes with 80 or better baseline BRVA was associated with a −12.81 ETDRS letter difference (95% CI −14.91, −10.71; *P* < 0.001) in BRVA at 2 years. Fellow eyes with baseline GA compared to absence had a mean difference of 3.35 ETDRS letters (95% CI 0.69, 6.01; *P* = 0.01) in BRVA in the study eye at 2 years. Study eyes with iRORA and SDD had reduced BRVA across all time points relative to eyes with no iRORA and no SDD in unadjusted analysis only (Supplementary Table S2), as this association was no longer evident after adjusting for age and gender (Table 3). Eyes with or without nAMD in the fellow-eye had no statistically significant difference in BRVA in the study eye across all time points.

**Table 3:** Adjusted mean difference in best recorded visual acuity (BRVA) over 2 years by study eye diagnosis, participant demographics and fellow eye status using linear mixed-effects models (LMEM’s)^a^.

	Baseline		M6		M12		M18		M24	
Characteristic	Difference (95% CI)	*P*-value	Difference (95% CI)	*P*-value	Difference (95% CI)	*P*-value	Difference (95% CI)	*P*-value	Difference (95% CI)	*P*-value
Age										
Per 1 year increase	−0.31 (−0.38, −0.25)	<0.001	−0.31 (−0.39, −0.24)	<0.001	−0.33 (−0.4, −0.26)	<0.001	−0.34 (−0.42, −0.26)	<0.001	−0.34 (−0.42, −0.25)	<0.001
Age, years										
<75	Ref		Ref		Ref		Ref		Ref	
75–84	−2.78 (−3.94, −1.63)	<0.001	−2.99 (−4.18, −1.8)	<0.001	−3.21 (−4.41, −2.01)	<0.001	−3.89 (−5.26, −2.52)	<0.001	−4.01 (−5.46, −2.55)	<0.001
85+	−6.5 (−8.27, −4.73)	<0.001	−6.81 (−8.63, −4.98)	<0.001	−7.03 (−8.88, −5.18)	<0.001	−5.92 (−8, −3.84)	<0.001	−5.82 (−8.05, −3.59)	<0.001
Sex										
F	Ref		Ref		Ref		Ref		Ref	
M	−0.38 (−1.51, 0.76)	0.52	−0.51 (−1.69, 0.66)	0.39	−0.14 (−1.33, 1.04)	0.81	−0.04 (−1.39, 1.3)	0.95	−0.31 (−1.75, 1.12)	0.67
Baseline BRVA, ETDRS letters [approximate Snellen]^b^										
*80 or better*										
*[20/25 or better]*	–	–	Ref		Ref		Ref		Ref	
*70–79*										
[20/40 to 20/25)	–	–	−6.52 (−7.51, −5.53)	<0.001	−7.08 (−8.1, −6.06)	<0.001	−6.66 (−7.85, −5.46)	<0.001	−7.34 (−8.59, −6.08)	<0.001
*≤69*										
*[worse than 20/40]*	–	–	−17.12 (−18.8, −15.44)	<0.001	−13.02 (−14.74, −11.29)	<0.001	−13.27 (−15.23, −11.3)	<0.001	−12.81 (−14.91, −10.71)	<0.001
iAMD diagnosis										
No atrophy & no SDD	Ref		Ref		Ref		Ref		Ref	
No atrophy & SDD	−0.3 (−1.54, 0.94)	0.64	0.31 (−0.97, 1.6)	0.63	0.49 (−0.81, 1.78)	0.46	0.87 (−0.61, 2.34)	0.25	−0.2 (−1.77, 1.36)	0.80
iRORA & no SDD	−0.12 (−1.76, 1.53)	0.89	−0.02 (−1.73, 1.69)	0.99	0.76 (−1.01, 2.53)	0.4	1.56 (−0.42, 3.54)	0.12	0.84 (−1.25, 2.93)	0.43
iRORA & SDD	−0.76 (−2.34, 0.82)	0.35	−1.21 (−2.88, 0.45)	0.15	−1.44 (−3.11, 0.22)	0.09	−1.37 (−3.25, 0.52)	0.16	−1.34 (−3.32, 0.65)	0.19
nAMD in fellow eye^c^										
Absent										
Present	−0.39 (−2.05, 1.26)	0.64	0.00 (−1.77, 1.76)	0.997	−0.48 (−2.23, 1.28)	0.59	−0.51 (−2.58, 1.56)	0.63	0.04 (−2.08, 2.16)	0.97
GA in fellow eye^c^										
Absent										
Present	1.9 (−0.18, 3.99)	0.07	2.19 (0.001, 4.38)	0.05	2.85 (0.66, 5.04)	0.01	3.21 (0.61, 5.81)	0.02	3.35 (0.69, 6.01)	.01

*iAMD* intermediate age-related macular degeneration, *iRORA* incomplete retinal and retinal pigment epithelial atrophy, *SDD* subretinal drusenoid deposits, *GA* Geographic atrophy, *ETDRS* Early treatment Diabetic Retinopathy Study, *BRVA* Best recorded visual acuity, *M*6 Month 6, *M*12 Month 12, *M*18 Month 18, *M*24 Month 24, *LMEM* Linear mixed-effects model.

^a^LMEM’s were fitted for the continuous outcome BRVA score employing an unstructured covariance structure to account for the within-participant correlation between repeated measures over time (baseline, month 6, month 12, month 18, month 24) and the nesting between eyes from the same participant. Fixed effects adjusted for include the main effects of the study eye diagnosis stage, fellow eye diagnosis, baseline BRVA, age and sex, and their interaction with time in separate LMEMs. The analysis presented here are adjusted for age and sex, with each variable additionally controlled for these covariates.

^b^For the variable baseline BRVA in the LMEM the outcome was not defined to include baseline time (follow-up time points to include M6, M12, M18 and M24). Eyes with missing baseline BRVA were not included, a total of *N* = 950 eyes from 782 participants were included for modelling this variable.

^c^For variables that indicate nAMD and GA in the fellow eye, nesting by eye was not considered, as only one eye per participant was included, and the random effects structure in LMEM was based solely on time.

### Change in BRVA: 10-letter and 15-letter loss at 2 years from baseline

A total of 935 eyes had data on BRVA at baseline and 2 years, of which 124/935 (13.3%) lost 10 or more letters and 58/935 (6.2%) lost 15 or more letters at 2 years from baseline (Table 4). Eyes with iRORA and no SDD had reduced odds of 10-letter BRVA loss (adjusted OR 0.42, 95% CI 0.18, 0.96, *P* = 0.039), compared to eyes with no iRORA and no SDD. At baseline presence of GA in the fellow eye was associated with reduced odds of 10-letter BRVA loss (adjusted OR 0.26, 95% CI 0.06, 0.72, *P* = 0.026). Male sex was associated with increased odds of 15-letter loss in BRVA by 2 years (age-adjusted OR 2.14, 95% CI 1.27, 3.63; *P* = 0.004, Table 4). Unadjusted logistic regression analysis is presented in Supplementary Table S3.

**Table 4:** Age- and sex-adjusted associations with 10-letter and 15-letter loss in best recorded visual acuity (BRVA) at 2 years from baseline estimated using logistic regression models via generalised estimating equations (GEE)^a^.

Baseline characteristic	*N*	<10 letters loss *N* = 811	≥10 letters loss *N* = 124	Adjusted OR (95% CI)	*p*-value	<15 letter loss *N* = 877	≥15 letter loss *N* = 58	Adjusted OR (95% CI)	*P*-value
Age, years, *n* (%)	935								
*<75*		366 (45.1%)	46 (37.1%)	—		392 (44.7%)	20 (34.5%)	Ref	–
*75–84*		349 (43.0%)	68 (54.8%)	1.57 (1.03–2.39)	0.037	384 (43.8%)	33 (56.9%)	1.62 (0.92–2.86)	0.09
*85*+		96 (11.8%)	10 (8.1%)	0.86 (0.41–1.79)	0.68	101 (11.5%)	5 (8.6%)	0.93 (0.34–2.57)	0.89
Age, years, mean (SD) or per 1 year increase	935	75.18(8.20)	76.27(6.66)	1.02 (1.00–1.04)	0.12	75.23 (8.14)	76.64 (5.79)	1.02 (0.99–1.05)	0.15
Sex	935								
*F*		541 (66.7%)	75 (60.5%)	—		588 (67.0%)	28 (48.3%)	Ref	–
*M*		270 (33.3%)	49 (39.5%)	1.29 (0.87–1.93)	0.21	289 (33.0%)	30 (51.7%)	2.14 (1.27–3.63)	0.004
Study eye diagnosis, *n*(%)	935								
No iRORA & no SDD		264 (32.6%)	45 (36.3%)	—		287 (32.7%)	22 (37.9%)	Ref	–
No iRORA & SDD		311 (38.3%)	51 (41.1%)	0.97 (0.62–1.52)	0.89	337 (38.4%)	25 (43.1%)	0.97 (0.53–1.78)	0.92
iRORA & no SDD		103 (12.7%)	7 (5.6%)	0.42 (0.18–0.96)	0.039	106 (12.1%)	4 (6.9%)	0.49 (0.16–1.46)	0.20
iRORA & SDD		133 (16.4%)	21 (16.9%)	0.99 (0.56–1.74)	0.96	147 (16.8%)	7 (12.1%)	0.64 (0.27–1.51)	0.31
Baseline BRVA, ETDRS letters [approximate Snellen], *n*(%)	935								
*80 or better [20/25 or better]*		543 (67.0%)	76 (61.3%)	—		587 (66.9%)	32 (55.2%)	Ref	–
*70–79 [20/40 to 20/25)*		206 (25.4%)	43 (34.7%)	1.43 (0.93–2.20)	0.10	226 (25.8%)	23 (39.7%)	1.82 (1.02–3.25)	0.04
*≤69 [worse than 20/40]*		62 (7.6%)	5 (4.0%)	0.64 (0.26–1.57)	0.33	64 (7.3%)	3 (5.2%)	0.89 (0.24–3.28)	0.86
Baseline BRVA, ETDRS letters, mean(SD) or per 1 letter increase	935	80(8)	80(6)	1.00 (0.98–1.02)	0.96	80(8)	79(6)	0.99 (0.97–1.01)	0.30
nAMD in the fellow eye, *n*(%)^b^	603								
*Absence*		105 (20.3%)	14 (16.5%)	—		112 (20.0%)	7 (16.7%)	Ref	–
*Presence*		413 (79.7%)	71 (83.5%)	1.25 (0.69–2.41)	0.47	449 (80.0%)	35 (83.3%)	1.22 (0.55–3.09)	0.64
GA in the fellow eye, *n*(%)^b^	603								
*Absence*		453 (87.5%)	82 (96.5%)			494 (88.1%)	41 (97.6%)	NA^c^	NA^c^
*Presence*		65 (12.5%)	3 (3.5%)	0.26 (0.06–0.72)	0.026	67 (11.9%)	1 (2.4%)		

*iAMD* intermediate age-related macular degeneration, *iRORA* incomplete retinal and retinal pigment epithelial atrophy, *SDD* subretinal drusenoid deposits, *GA* Geographic atrophy, *BRVA* Best recorded visual acuity, *GEE* Generalised estimating equations, *OR* Odds Ratio.

^a^Adjusted logistic regression models via GEE using geeglm() function from the geepack package in R was used for modelling binary outcomes 10-letter and 15-letter loss at 2 years from baseline. Age-sex adjusted OR’s with 95% CI are presented.

^b^Logistic regression models were fit using glm() function in R without the use of GEE, as only a single eye was included (eyes with unilateral eligibility).

^c^Insufficient sample size for modelling, so the model for GA in the fellow eye as a variable was not fitted.

### Sensitivity analysis

In sensitivity analysis with BCVA as the outcome, there was negligible difference in estimated marginal mean BCVA over time compared to estimated marginal mean BRVA (Supplementary Fig. S1). The estimated difference in BCVA at each time point was −0.36 ETDRS letters (95% CI −0.87, 0.15); *P* = 0.16 at 6 months, −0.69 ETDRS letters (95% CI −1.26, −0.11); *P* = 0.02 at 12 months, −1.59 ETDR letters (95% CI −2.31, −0.88); *P* < 0.001 at 18 months and −2.35 ETDRS letters (95% CI −3.13, −1.57); *P* < 0.001 at 24 months compared to baseline. By month 24, 132 eyes had progressed; 53 (5.4%) had progressed to GA and 79 (8.0%) to nAMD. After excluding study eyes that developed nAMD or GA during the study, there was negligible difference in estimated marginal mean BRVA over time (Supplementary Fig. S2). The estimated difference in BRVA at each time point after excluding eyes that developed nAMD or GA during the study was −0.33 ETDRS letters (95% CI −0.72, 0.06); *P* = 0.056 at 6 months, −0.53 ETDRS letters (95% CI −1.02, −0.04); *P* = 0.03 at 12 months, −1.06 ETDRS letters (95% CI −1.61, −0.51); *P* < 0.001 at 18 months and −1.56 ETDRS letters (95% CI −2.17, −0.95); *P* < 0.001 at 24 months. Additionally, eyes with missing BRVA over time were similar in distribution in baseline BRVA and study eye diagnoses to those with available data (Supplementary Table S4).

## Discussion

This study demonstrates that individuals with iAMD show minimal change in BRVA with an average loss of ~2 ETDRS letters over 2 years in the real-world irrespective of whether routinely collected BRVA or BCVA is used in the analysis. In the repeatability study within the MACUSTAR prospective cohort, the mean deviation of BCVA for each AMD group (early, intermediate and late AMD) was also within 2 ETDRS letters [12]. Data from the AREDS2 report of 1105 nAMD eyes treated with antiVEGF in routine practice showed that vision declined by ~1.5–2 letters per year, thus 3 ~ 4 letters lost after 2 years [13]. Similarly, data from the COMPLETE trial in GA eyes showed an average loss of 2.9 letters in the placebo group compared with 0.7 letters in the eculizumab group at 1 year [14]. These findings align with expected disease trajectories, as our results show slightly less visual decline than typically observed in GA and nAMD cohorts, consistent with the more progressive and rapid VA deterioration seen in later stage AMD. About 90% of individuals in our study presented with BRVA 70 letters or better and around 65% with at least 80 letters and so most individuals with iAMD have good vision creating a ceiling effect and therefore are more prone to lose rather than gain in VA. Only 13% had a 10 or more-letter loss by 2 years and 7% had 15 or more-letter loss by 2 years, both accepted criterion used to define a minimum clinically meaningful change in AMD and retinal vascular diseases. These results reinforce the fact that a prevention of 15-letter loss is not an achievable endpoint in iAMD if the control arm has a mean loss of only 2 letters over two years and very few eyes lost 15 or more letters [4].

These results also highlight the importance of baseline VA when evaluating and comparing clinical trials in iAMD. Those presenting with less than 70 letters, also had lower BRVA at the 2 year mark compared to those presenting with BRVA of at least 80 letters (adjusted difference of −12.81 ETDRS letters). It is also well-established that lower VA present with greater variability and has a floor effect. Therefore, including patients with iAMD and BRVA less than 70 letters in clinical trials may increase the likelihood of unexpected results especially if the intervention and control arms are not balanced for visual acuity. In addition, these findings may be interpreted as regression to the mean too.

Another factor to consider is that increasing age at baseline was linked to lower BRVA across all time points (6, 12, 18 and 24 months), with a reduction of 0.34 letters per year of baseline age at 24 months. Individuals aged 75–84 and those ≥85 years had lower BRVA outcome at all time points across the 2 years compared to <75 age group. Multiple age-related ocular changes including increasing thickness of the Bruch’s membrane, lipid accumulation, drusen volume and decreasing density of choriocapillaris may explain this observation [15, 16]. The changes may also be confounded by non-AMD related BRVA decline with time. Moreover, we found that male sex was associated with loss of 15 or more letters in adjusted analyses. While no study in iAMD has found such associations, this is consistent with the CATT trial which demonstrated that female participants were significantly more likely to gain 15 or more letters by 5 years [17].

Interestingly, based on our classification of iAMD phenotypes, all groups showed a mean decline in VA over two years with no difference in mean change between them. After excluding study eyes that developed nAMD or GA during the study, there was negligible difference in estimated marginal mean BRVA over time. Although eyes with SDD (both with and without iRORA) presented with lower BRVA and remained so at two years, the estimated difference in mean BRVA at 2 years did not reach statistical significance, in contrast to previous reports from smaller studies [18]. On the contrary, by 2 years, eyes with iRORA but no SDD were less common among those with ≥10- and ≥15-letter loss. Specifically, only ~6% of eyes with ≥10-letter loss and ~7% with ≥15-letter loss had this phenotype, compared to more than 10% among those without such losses. Though the iRORA without SDD phenotype was significantly associated with lower odds of 10-letter loss (adjusted OR 0.42, 95% CI 0.18–0.96, *P* = 0.039), but not with 15-letter loss, likely due to limited sample size. These findings highlight that although iRORA is a risk factor for transition to cRORA, this structural precursor of atrophy is not associated with VA decline. Moreover, these findings may also be because iRORA may be reversible [19].

Our results also show that study eyes with GA in the fellow eye had reduced odds of 10-letter loss in BRVA by 2 years; there was insufficient sample size to study associations with 15-letter loss. This may demonstrate the slow course of the transition from iAMD to atrophy without substantial VA loss. In fact, the presence of GA in the fellow eye was associated with an improved BRVA outcome in the study eye across all time points, with a mean difference of +3.35 letters at month 24 relative to study eyes without GA in the fellow eye. This finding raises the question whether participants with fellow eye GA attempts to read better at each visit than those without GA in the fellow eye. Those within 1-line changes in BRVA in the natural course of iAMD has been confirmed in other studies and should also be considered when interpreting clinical trials in iAMD [20, 21]. While our findings indicate that fellow eyes of patients with unilateral nAMD or GA may be prime candidates for clinical trials of preventive interventions, eyes with bilateral iAMD whom still experience losses in vision are underrepresented relative to the general population of older adults and inclusion of bilateral iAMD can improve generalisability and completeness of findings.

Although this is a large cohort with iAMD, the study has some limitations. The classification of eyes as presence or absence of SDD may be imperfect as the quantity or area of SDD may need to be incorporated for finer phenotyping [22]. Furthermore, the definition of iAMD assumes soft drusen are distributed in the macula and other risk characteristics of drusen such as hyporeflective drusen, cuticular drusen and drusenoid pigment epithelial detachment may also influence disease progression [23, 24]. A more detailed grading of iAMD may elicit functional changes not revealed in this study [21]. However, in our study the key objective was to analyse the trajectory of VA in a large iAMD cohort and compare these trajectories across key subgroups of iAMD diagnosis stage. To mitigate bias due to potential confounding, we adjusted for relevant baseline demographic variables. Finally, the method of VA measurement varied across visits for each participant (best-corrected, with glasses or pinhole) which represents a potential limitation when analysing small changes in VA over time, however we also report a sensitivity analysis restricted to BCVA at each time point to show the trajectory of change over the two-year period, with negligible differences.

In conclusion, eyes with iAMD experience a small mean decline of approximately 2 letters in BRVA over two years. While these changes may not represent a clinically meaningful difference, the findings of this large cohort study provide insights for further research on VA change in iAMD especially for designing future clinical trials.

## Summary

### What was known before

- Change in visual acuity (VA) from baseline is an established outcome measure for intervention trials in age-related macular degeneration.
- For regulatory approval, a preferred outcome is a prevention or improvement in VA of at least 15 Early Treatment Diabetic Retinopathy Study (ETDRS) letters (a change of doubling of the visual angle).
- Eyes with intermediate AMD present with good visual acuity.
- Minimal changes in VA are expected in iAMD over time but the baseline determinants of VA loss are unclear.

### What this study adds

- The average loss of best recorded visual acuity (BRVA) in iAMD is only 2 ETDRS letters by two years and those with higher baseline BRVA and increasing age are more likely to lose BRVA, although the change is not clinically meaningful.
- Eyes with baseline BRVA of worse than 70 ETDRS letter score had a -12-letter difference in BRVA at the 2-year mark compared to eyes that presented with 80 ETDRS letter score or more. Therefore, it is advisable that clinical trials on iAMD stratify participants by baseline BRVA.
- Only 7% of the study cohort lost 15 or more letters so preventing a 15-letter loss in this group by any intervention in eyes with iAMD require large sample size and longer follow-up than two years if the primary outcome is change in BRVA.
- GA in the fellow eye was less common among eyes that experience BRVA loss, where the odds of 10-letter loss by 2 years was reduced by a factor of ~3.85 compared to absence of GA.
- Males with iAMD had more than twice the odds of losing 15 or more letters by 2 years.

## Supplementary information

Table S1. Baseline summary statistics by study eye diagnosis

Table S2. Unadjusted mean difference in best recorded visual acuity (BRVA) over 2 years by study eye diagnosis, demographics and fellow eye status using linear mixed-effects models (LMEM’s)a

Table S3. Univariate analysis for 10- and 15-letter losers in best recorded visual acuity (BRVA) at 2 years from baseline, using logistic regression models via generalized estimating equations (GEE)a

Table S4. Comparing baseline characteristics in eyes with missing or complete data in best recorded visual acuity (BRVA) across all follow-up time points

Table S5. INTERCEPT-AMD Study Group

Figure S1. Sensitivity analysis on the natural history of best corrected visual acuity (BCVA) as the outcome instead of best recorded visual acuity (BRVA) using linear mixed-effects models (LMEM’s)a

Figure S2. Sensitivity analysis on best recorded visual acuity (BRVA) natural history, setting BRVA to missing in eyes that converted to nAMD or GA using linear mixed effects models (LMEM's)a

## Data Availability

The data that support the findings of this study are all reported in this paper. Further details are available from the corresponding author and/or in the EVICR.net Eye Platform upon reasonable request.
