# Supplementary material for: Baseline factors that are associated with change in visual acuity in intermediate AMD over two years in a multicentre cohort study in Europe- INTERCEPT-AMD Report 2
Source: Eye (Lond). 2025 Oct 17;39(18):3324–32. doi: 10.1038/s41433-025-04062-z (PMC12669714; doi:10.1038/s41433-025-04062-z)
Supplement: Supplementary file 1 — Table S1. Baseline summary statistics by study eye diagnosis [file 41433_2025_4062_MOESM1_ESM.docx]

Table S1. Baseline summary statistics by study eye diagnosis

| Baseline characteristic | Overall, N=**983** eyes of **805** participants | no atrophy & no SDD, N = 321 eyes of 273 participants | no atrophy with SDD, N = 385 eyes of 323 participants | iRORA & no SDD, N = 114 eyes of 106 participants | iRORA with SDD, N = 163 eyes of 142 participants |
| --- | --- | --- | --- | --- | --- |
| Participant level (N=**805** participants) |  |  |  |  |  |
| Age, years | 75.8 (7.9) | 74.0 (7.8) | 77.1 (7.5) | 72.8 (8.5) | 78.0 (7.6) |
| Age, years, categories |  |  |  |  |  |
| <75 | 333 (41.4%) | 138 (50.5%) | 114 (35.3%) | 61 (57.5%) | 40 (28.2%) |
| 75-84 | 371 (46.1%) | 116 (42.5%) | 159 (49.2%) | 39 (36.8%) | 74 (52.1%) |
| 85+ | 101 (12.5%) | 19 (7.0%) | 50 (15.5%) | 6 (5.7%) | 28 (19.7%) |
| Sex |  |  |  |  |  |
| F | 523 (65.0%) | 168 (61.5%) | 213 (65.9%) | 62 (58.5%) | 102 (71.8%) |
| M | 282 (35.0%) | 105 (38.5%) | 110 (34.1%) | 44 (41.5%) | 40 (28.2%) |
| Bilaterality, % | 178 (22.1%) | 48 (17.6%) | 62 (19.2%) | 8 (7.6%) | 21 (14.8%) |
| AMD category, fellow eye |  |  |  |  |  |
| Bilateral eligibility | 178 (22.1%) | 48 (17.6%) | 62 (19.2%) | 8 (7.5%) | 21 (14.8%) |
| Not recorded or insufficient data | 8 (1.0%) | 21 (7.7%) | 30 (9.3%) | 19 (17.9%) | 16 (11.3%) |
| Early AMD fellow eye | 11 (1.4%) | 5 (1.8%) | 2 (0.6%) | 2 (1.9%) | 2 (1.4%) |
| Established Geographic Atrophy (cRORA) fellow eye | 72 (8.9%) | 15 (5.5%) | 18 (5.6%) | 17 (16.0%) | 22 (15.5%) |
| nAMD (presence of MNV) fellow eye | 499 (62.0%) | 160 (58.6%) | 202 (62.5%) | 59 (55.7%) | 78 (54.9%) |
| Healthy macula fellow eye | 5 (0.6%) | 3 (1.1%) | 2 (0.6%) | 0 (0%) | 0 (0%) |
| Other retinal disease than AMD related in fellow eye | 32 (4.0%) | 21 (7.7%) | 7 (2.2%) | 1 (0.9%) | 3 (2.1%) |
| Eye level (N=**983** eyes) |  |  |  |  |  |
| BRVA, study eye, ETDRS letters, mean (SD) [median (IQR)] | 79.8 (8.1) [80.0 (75.0, 85.0)] | 80.3 (7.8)  [81.0 (75.0, 85.0)] | 79.5(8.5)  [80.0 (75.0, 85.0)] | 80.5(7.4)  [83.0 (75.0, 85.0)] | 78.9(8.2)  [80.0 (75.0, 85.0)] |
| Missing | 33 | 6 | 18 | 4 | 5 |
| BRVA, study eye, ETDRS letters [approximate Snellen], categories |  |  |  |  |  |
| <37  [worse than 20/200] | 3 (0.32%) | 1 (0.3%) | 2 (0.5%) | 0 (0.0%) | 0 (0.0%) |
| 37-54  [20/200 to 20/80) | 9 (0.95%) | 3 (1.0%) | 3 (0.8%) | 1 (0.9%) | 2 (1.3%) |
| 55-69  [20/80 to 20/50) | 56 (5.89%) | 17 (5.4%) | 25 (6.8%) | 5 (4.5%) | 9 (5.7%) |
| 70-79  [20/50 to 20/25) | 252 (26.5%) | 73 (23.2%) | 95 (25.9%) | 31 (28.2%) | 53 (33.5%) |
| 80 or better  [20/25 or better] | 630 (66.3%) | 221 (70.2%) | 242 (65.9%) | 73 (66.4%) | 94 (59.5%) |
| Missing | 33 | 6 | 18 | 4 | 5 |
| VA method, study eye, % |  |  |  |  |  |
| Missing | 36 (3.66%) | 6 (1.9%) | 20 (5.2%) | 4 (3.5%) | 6 (3.7%) |
| Best corrected | 634 (64.5%) | 203 (63.2%) | 273 (70.9%) | 66 (57.9%) | 92 (56.4%) |
| Glasses | 305 (31.0%) | 110 (34.3%) | 88 (22.9%) | 42 (36.8%) | 65 (39.9%) |
| Pinhole | 8 (0.81%) | 2 (0.6%) | 4 (1.0%) | 2 (1.8%) | 0 (0.0%) |
| Total line scans |  |  |  |  |  |
| <49 | 689 (70.1%) | 217 (67.6%) | 281 (73.0%) | 88 (77.2%) | 103 (63.2%) |
| >=49 | 294 (29.9%) | 104 (32.4%) | 104 (27.0%) | 26 (22.8%) | 60 (36.8%) |

Abbreviations: iAMD-intermediate age-related macular degeneration; cRORA- complete retinal and retinal pigment epithelial atrophy; iRORA- incomplete retinal and retinal pigment epithelial atrophy; MNV-macular neovascularisation; SDD-subretinal drusenoid deposits; BRVA-Best recorded visual acuity; ETDRS-Early treatment Diabetic Retinopathy Study; GA-geographic atrophy.
