# Supplementary material for: Baseline factors that are associated with change in visual acuity in intermediate AMD over two years in a multicentre cohort study in Europe- INTERCEPT-AMD Report 2
Source: Eye (Lond). 2025 Oct 17;39(18):3324–32. doi: 10.1038/s41433-025-04062-z (PMC12669714; doi:10.1038/s41433-025-04062-z)
Supplement: Supplementary file 2 — Table S2. Unadjusted mean difference in best recorded visual acuity (BRVA) over 2 years by study eye diagnosis, demographics and fellow eye status using linear mixed-effects models (LMEM’s)a [file 41433_2025_4062_MOESM2_ESM.docx]

Table S2. Unadjusted mean difference in best recorded visual acuity (BRVA) over 2 years by study eye diagnosis, participant demographics and fellow eye status using linear mixed-effects models (LMEM’s)^a^

|  | Baseline | | M6 | | M12 | | M18 | | M24 | |
| --- | --- | --- | --- | --- | --- | --- | --- | --- | --- | --- |
| Characteristic | Difference (95% CI) | P-value | Difference (95% CI) | P-value | Difference (95% CI) | P-value | Difference (95% CI) | P-value | Difference (95% CI) | P-value |
| **Age, years** |  |  |  |  |  |  |  |  |  |  |
| Per 1 year increase | -0.31(-0.38,-0.25) | **<.001** | -0.32(-0.39,-0.25) | **<.001** | -0.33(-0.4,-0.26) | **<.001** | -0.34(-0.42,-0.26) | **<.001** | -0.34(-0.42,-0.25) | **<.001** |
| **Age, years** |  |  |  |  |  |  |  |  |  |  |
| **<75** | Ref |  | Ref |  | Ref |  | Ref |  | Ref |  |
| 75-84 | -2.79(-3.94,-1.63) | **<.001** | -3.04(-4.22,-1.85) | **<.001** | -3.22(-4.42,-2.01) | **<.001** | -3.88(-5.24,-2.51) | **<.001** | -4.00(-5.45,-2.54) | **<.001** |
| 85+ | -6.5(-8.28,-4.73) | **<.001** | -6.84(-8.66,-5.01) | **<.001** | -7.05(-8.9,-5.2) | **<.001** | -5.92(-8,-3.83) | **<.001** | -5.82(-8.06,-3.59) | **<.001** |
| **Sex** |  |  |  |  |  |  |  |  |  |  |
| **F** | Ref |  | Ref |  | Ref |  | Ref |  | Ref |  |
| M | -0.47(-1.65,0.71) | .44 | -0.62(-1.84,0.6) | .32 | -0.23(-1.46,1) | .72 | -0.13(-1.52,1.25) | .85 | -0.4(-1.86,1.07) | .60 |
| **Baseline BRVA, ETDRS letters [approximate Snellen]^b^** |  |  |  |  |  |  |  |  |  |  |
| *80 or better [20/25 or better]* | - | - | Ref |  | Ref |  | Ref |  | Ref |  |
| *70-79 [20/40 to 20/25)* | - | - | -7.02(-8,-6.04) | **<.001** | -7.55(-8.57,-6.52) | **<.001** | -7.1(-8.29,-5.91) | **<.001** | -7.78(-9.04,-6.53) | **<.001** |
| *<=69 [worse than 20/40]* | - | - | -17.77(-19.44,-16.09) | **<.001** | -13.65(-15.38,-11.92) | **<.001** | -13.89(-15.86,-11.92) | **<.001** | -13.47(-15.57,-11.37) | **<.001** |
| **iAMD diagnosis** |  |  |  |  |  |  |  |  |  |  |
| No atrophy & no SDD | Ref |  | Ref |  | Ref |  | Ref |  | Ref |  |
| No atrophy & SDD | -0.99(-2.25,0.26) | .12 | -0.38(-1.7,0.93) | .57 | -0.27(-1.59,1.05) | .69 | 0.11(-1.38,1.6) | .88 | -0.98(-2.56,0.59) | .22 |
| iRORA & no SDD | 0.03(-1.64,1.71) | .97 | 0.16(-1.59,1.91) | .86 | 0.89(-0.92,2.7) | .33 | 1.69(-0.32,3.71) | .099 | 0.96(-1.16,3.08) | .38 |
| iRORA & SDD | -1.77(-3.36,-0.18) | **.03** | -2.31(-3.99,-0.62) | **.007** | -2.54(-4.22,-0.86) | **.003** | -2.5(-4.4,-0.6) | **.01** | -2.46(-4.46,-0.47) | **.02** |
| **nAMD in fellow eye^c^** |  |  |  |  |  |  |  |  |  |  |
| Absent | Ref |  | Ref |  | Ref |  | Ref |  | Ref |  |
| Present | -0.83(-2.52,0.87) | .34 | -0.5(-2.3,1.29) | .58 | -0.9(-2.7,0.9) | .33 | -0.99(-3.1,1.12) | .36 | -0.4(-2.55,1.76) | .72 |
| **GA in fellow eye^c^** |  |  |  |  |  |  |  |  |  |  |
| Absent | Ref |  | Ref |  | Ref |  | Ref |  | Ref |  |
| Present | 2.17(0.04,4.3) | **.046** | 2.48(0.24,4.72) | **.03** | 3.14(0.88,5.39) | **.006** | 3.51(0.87,6.15) | **.009** | 3.63(0.93,6.34) | **.009** |

Abbreviations: iAMD-intermediate age related macular degeneration; iRORA- incomplete retinal and retinal pigment epithelial atrophy; SDD-subretinal drusenoid deposits; ETDRS-Early treatment Diabetic Retinopathy Study; BRVA-best recorded visual acuity; GA-geographic atrophy; LMEM-Linear mixed-effects models

^a^ LMEM’s were fitted for the continuous outcome BRVA score employing an unstructured covariance structure to account for the within-participant correlation between repeated measures over time (baseline, month 6, month 12, month 18, month 24) and the nesting between eyes from the same participant. Fixed effects adjusted for include the main effects of the study eye diagnosis stage, fellow eye diagnosis, age and sex, and their interaction with time in separate univariate LMEM’s.

^b^ For the variable baseline BRVA, in the LMEM the outcome was not defined to include baseline time (follow-up time included months 6, 12, 18 and 24). Eyes with missing baseline BRVA were not included, a total of N=950 eyes from 782 participants were included for modelling.

^c^ For variables that indicate nAMD and GA in the fellow eye, nesting by eye was not considered, as only one eye per participant was included, and the random effects structure in LMEM’s was based solely on time
