# Supplementary material for: Baseline factors that are associated with change in visual acuity in intermediate AMD over two years in a multicentre cohort study in Europe- INTERCEPT-AMD Report 2
Source: Eye (Lond). 2025 Oct 17;39(18):3324–32. doi: 10.1038/s41433-025-04062-z (PMC12669714; doi:10.1038/s41433-025-04062-z)
Supplement: Supplementary file 3 — Table S3. Univariate analysis for 10- and 15-letter losers in best recorded visual acuity (BRVA) at 2 years from baseline, using logistic regression models via generalized estimating equations (GEE)a [file 41433_2025_4062_MOESM3_ESM.docx]

|  |  | **>=10 letter loss** |  | **>=15 letter loss** |  |
| --- | --- | --- | --- | --- | --- |
| **Baseline characteristic** | **N** | **Unadjusted OR (95% CI)** | **P-value** | **Unadjusted OR (95% CI)** | **P-value** |
| **Age, years, n(%)** | 935 |  |  |  |  |
| *<75* |  | — |  | Ref | - |
| *75-84* |  | 1.58 (1.04 - 2.41) | **0.034** | 1.68 (0.95 - 2.97) | .07 |
| *85+* |  | 0.87 (0.42 - 1.80) | 0.70 | 0.96 (0.35 - 2.64) | .94 |
| **Age, years, mean (SD) or per 1 year increase** | 935 | 1.02 (1.00 - 1.04) | 0.10 | 1.02 (1.00 - 1.05) | .09 |
| **Sex** | 935 |  |  |  |  |
| *F* |  | — |  | Ref | - |
| *M* |  | 1.31 (0.88 - 1.95) | 0.19 | 2.18 (1.29 - 3.71) | **.004** |
| **Study eye diagnosis, n(%)** | 935 |  |  |  |  |
| No iRORA & no SDD |  | — |  | Ref | - |
| No iRORA & SDD |  | 0.97 (0.62 - 1.51) | 0.89 | 0.97 (0.54 - 1.74) | .91 |
| iRORA & no SDD |  | 0.41 (0.18 - 0.95) | **0.037** | 0.49 (0.16 - 1.46) | .20 |
| iRORA & SDD |  | 0.97 (0.55 - 1.69) | 0.91 | 0.62 (0.26 - 1.48) | .28 |
| **Baseline BRVA, ETDRS letters [approximate Snellen], n(%)** | 935 |  |  |  |  |
| *80 or better [20/25 or better]* |  | — |  | Ref | - |
| *70-79 [20/40 to 20/25)* |  | 1.48 (0.98 - 2.25) | 0.063 | 1.87 (1.07 - 3.26) | **.03** |
| *<=69 [worse than 20/40]* |  | 0.62 (0.25 - 1.50) | 0.29 | 0.85 (0.25 - 2.90) | .80 |
| **Baseline BRVA, ETDRS letters, mean(SD) or per 1 letter increase** | 935 | 1.00 (0.98 - 1.02) | 0.82 | 0.99 (0.97 - 1.01) | .19 |
| **nAMD in the fellow eye, n(%)^b^** | 603 |  |  |  |  |
| *Absence* |  | — |  | Ref | - |
| *Presence* |  | 1.29 (0.72 - 2.47) | 0.42 | 1.25 (0.57 - 3.13) | .61 |
| **GA in the fellow eye, n(%)^b^** | 603 |  |  |  |  |
| *Absence* |  | — |  | NA^c^ | NA^c^ |
| *Presence* |  | 0.25 (0.06 - 0.71) | **0.023** |  |  |

Table S3. Univariate analysis for 10- and 15-letter losers in best recorded visual acuity (BRVA) at 2 years from baseline estimated using logistic regression models via generalized estimating equations (GEE)^a^

Abbreviations: iAMD-intermediate age related macular degeneration; iRORA- incomplete retinal and retinal pigment epithelial atrophy; SDD-subretinal drusenoid deposits; GA-Geographic atrophy; BRVA-Best recorded visual acuity; GEE-Generalised estimating equations; OR- Odds Ratio.

^a^ Adjusted logistic regression via GEE using geeglm() function from the geepack package in R was used for modelling binary outcomes 10-letter and 15-letter loss at 2 years from baseline. Unadjusted OR’s with 95% CI are presented.

^b^ Logistic regression models were fit using glm() function in R without the use of GEE, as only a single eye was included (eyes with unilateral eligibility)

^c^ Insufficient sample size for modelling, so the model for GA in the fellow eye as a variable was not fitted
