# Supplementary material for: Baseline factors that are associated with change in visual acuity in intermediate AMD over two years in a multicentre cohort study in Europe- INTERCEPT-AMD Report 2
Source: Eye (Lond). 2025 Oct 17;39(18):3324–32. doi: 10.1038/s41433-025-04062-z (PMC12669714; doi:10.1038/s41433-025-04062-z)
Supplement: Supplementary file 4 — Table S4. Comparing baseline characteristics in eyes with missing or complete data in best recorded visual acuity (BRVA) across all follow-up time points [file 41433_2025_4062_MOESM4_ESM.docx]

Table S4. Comparing baseline characteristics in eyes with missing or complete data in best recorded visual acuity (BRVA) across all follow-up time points

| **Baseline characteristic** | **Eyes with missing / non-missing data in BRVA across time** | | | | | | | |
| --- | --- | --- | --- | --- | --- | --- | --- | --- |
|  | **6 months** | | **12 months** | | **18 months** | | **24 months** | |
|  | **Missing**  **(N=338)** | **Non-missing**  **(N=645)** | **Missing**  **(N=122)** | **Non-missing**  **(N=861)** | **Missing**  **(N=374)** | **Non-missing**  **(N=609)** | **Missing**  **(N=22)** | **Non-missing**  **(N=961)** |
| **BRVA, ETDRS letters [approximate Snellen], categories, n(%)**  <=69 [worse than 20/40]  70-79 [20/40 to 20/25)  80 or better [20/25 or better] | 24 (7.5%)  89 (27.9%)  206 (64.6%)  [N=319] | 44(7.0%)  163(25.8%)  424 (67.2%)  [N=631] | 9 (8.3%)  30 (27.8%)  69 (63.9%)  [N=108] | 59 (7.0%)  222 (26.4%)  561 (66.6%)  [N=842] | 23 (6.4%)  97 (27.0%)  239 (66.6%)  [N=359] | 45 (7.6%)  155 (26.2%)  391 (66.2%)  [N=591] | 1 (6.7%)  3 (20.0%)  11 (73.3%)  [N=15] | 67 (7.2%)  249 (26.6%)  619 (66.2%)  [N=935] |
| **BRVA, ETDRS letters, mean(SD)** | 79.8 (8.5)  [N=319] | 79.8 (7.9)  [N=631] | 79.0 (8.6)  [N=108] | 79.9 (8.0)  [N=842] | 80.2 (7.2)  [N=359] | 79.6 (8.6)  [N=591] | 78.4(10.2)  [N=15] | 79.8 (8.1)  [N=935] |
| **iAMD phenotype, categories, n(%)**  No iRORA & no SDD  No iRORA & SDD  iRORA & no SDD  iRORA & SDD | 123 (36.4%)  117 (34.6%)  31 (9.2%)  67 (19.8%) | 198 (30.7%)  268 (41.6%)  83 (12.9%)  96 (14.9%) | 35 (28.7%)  44 (36.1%)  18 (14.8%)  25 (20.5%) | 86 (33.2%)  341 (39.6%)  96 (11.1%)  138 (16.0%) | 142 (38.0%)  124 (33.2%)  41 (11.0%)  67 (17.9%) | 179 (29.4%)  261 (42.9%)  73 (12.0%)  96 (15.8%) | 6 (27.3%)  11 (50.0%)  0 (0.0%)  5 (22.7%) | 315 (32.8%)  374 (38.9%)  114 (11.9%)  158 (16.4%) |

Abbreviations: iAMD-intermediate age related macular degeneration; iRORA- incomplete retinal and retinal pigment epithelial atrophy; SDD-subretinal drusenoid deposits; ETDRS-Early treatment Diabetic Retinopathy Study; BRVA-best recorded visual acuity
