# Supplementary material for: Baseline factors that are associated with change in visual acuity in intermediate AMD over two years in a multicentre cohort study in Europe- INTERCEPT-AMD Report 2
Source: Eye (Lond). 2025 Oct 17;39(18):3324–32. doi: 10.1038/s41433-025-04062-z (PMC12669714; doi:10.1038/s41433-025-04062-z)
Supplement: Supplementary file 5 — Table S5. INTERCEPT-AMD Study Group [file 41433_2025_4062_MOESM5_ESM.docx]

**Table S5. Intercept-AMD Study Group**

| **Principal Investigator** | **Affiliations** |
| --- | --- |
| Inês Marques | Centre for Clinical Trials . AIBILI / Association for Innovation and Biomedical Research on Light and Image Coimbra, Portugal |
| Jean-François Girmens | Centre d’Investigation Clinique Centre National d’Ophtalmologie des Quinze-Vingts Paris, France |
| Sobha Sivaprasad | NIHR Moorfields Clinical Research Facility, Moorfields Eye Hospital, NHS Foundation Trust London, UK |
| Yara Lechanteur | Department of Ophthalmology  Radboud University Medical Centre Nijmegen, Netherlands |
| Mariacristina Parravano | IRCCS Fondazione G.B. Bietti per lo Studio e la Ricerca in Oftalmologia ONLUS Rome, Italy  UniCamillus-Saint Camillus International University of Health Sciences, Rome, Italy |
| Lieselotte Berger | Department of Ophthalmology, Inselspital, University of Bern Bern, Switzerland |
| Hansjürgen Agostini | Department of Ophthalmology University of Freiburg Freiburg, Germany |
| Sandra Barrão | Instituto de Oftalmologia Dr. Gama Pinto Lisboa, Portugal |
| Evangelos Tsiroukis | Institut Català de Retina (ICR), Clinical Trial Unit, Barcelona, Spain |
| Jordi Monés | Institut de la Màcula  Centro Médico Teknon Barcelona, Spain |
| Laura Sararols | Valles Ophthalmology Research, S.L. Barcelona, Spain |
| Rufino Silva | (1) Faculty of Medicine. University of Coimbra. Portugal  (2) Ophthalmology Department. Coimbra University Hospital. ULS Coimbra. Portugal  (3) Association for Innovation and Biomedical Research on Light and Image (AIBILI). Coimbra. Portugal |
| Hendrik P.N. Scholl | (1) Medical University of Vienna, Department of Clinical Pharmacology, Vienna, Austria  (2) Pallas Kliniken AG, Pallas Klinik Zürich, Zürich, Switzerland  (3) European Vision Institute, Basel, Switzerland |
| Albrecht Lommatzsch | Department of Ophthalmology St. Franziskus-Hospital Münster, Germany |
| Boris Stanzel | Eye Clinic Sulzbach, Knappschaft Hospital Saar Sulzbach, Germany |
| Stela Vujosevic | Medical Retina Unit, Eye Clinic- IRCCS MultiMedica, MultiMedica Milan, Italy,  University of Milan, Milan, Italy |
| Yannick Liermann | Department of Ophthalmology University of Bonn, Bonn, Germany |
| Paolo Lanzetta | Department of Ophthalmology University of Udine, Udine, Italy |
| Emily Fletcher | Clinical Trial Unit, Dep. Ophth., Gloucestershire Hospitals NHS Foundation Trust, Cheltenham, UK |
| Savita Madhusudhan | Clinical Eye Research Centre – St. Paul’s Eye Unit, Royal Liverpool University Hospital, Liverpool, UK |
| Lyubomyr Lytvynchuk | Department of Ophthalmology, Justus-Liebig-University-Giessen Giessen, Germany |
| Francesco Bandello | Department of Ophthalmology, University Vita Salute - Scientific Institute of San Raffaele Milan, Italy |
| Nicole Eter | Department of Ophthalmology, University of Muenster Medical Center, Münster, Germany |
| Stefano de Cilla | Eye Unit, University Hospital Maggiore della Carità, Novara, Italy |
| Michel Weber | Department of Ophthalmology, University Hospital, Nantes, France |
| Aude Ambresin | Swiss Visio Retina Research Center, Swiss Visio Montchoisi, Lausanne, Switzerland |
