# Supplementary figures and images for: Baseline factors that are associated with change in visual acuity in intermediate AMD over two years in a multicentre cohort study in Europe- INTERCEPT-AMD Report 2
Source: Eye (Lond). 2025 Oct 17;39(18):3324–32. doi: 10.1038/s41433-025-04062-z (PMC12669714; doi:10.1038/s41433-025-04062-z)

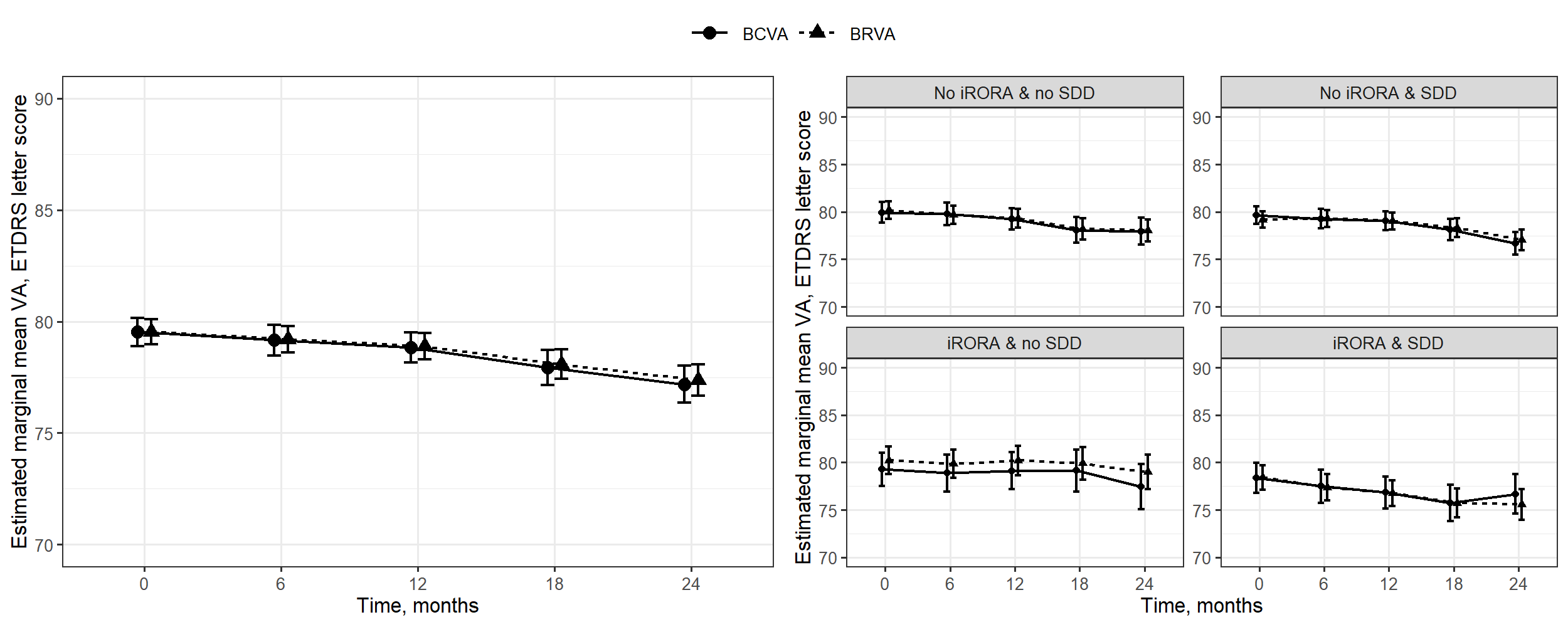

Supplement: Supplementary file 6 — Figure S1. Sensitivity analysis on the natural history of best corrected visual acuity (BCVA) as the outcome instead of best recorded visual acuity (BRVA) using linear mixed-effects models (LMEM’s)a [file 41433_2025_4062_MOESM6_ESM.tif]

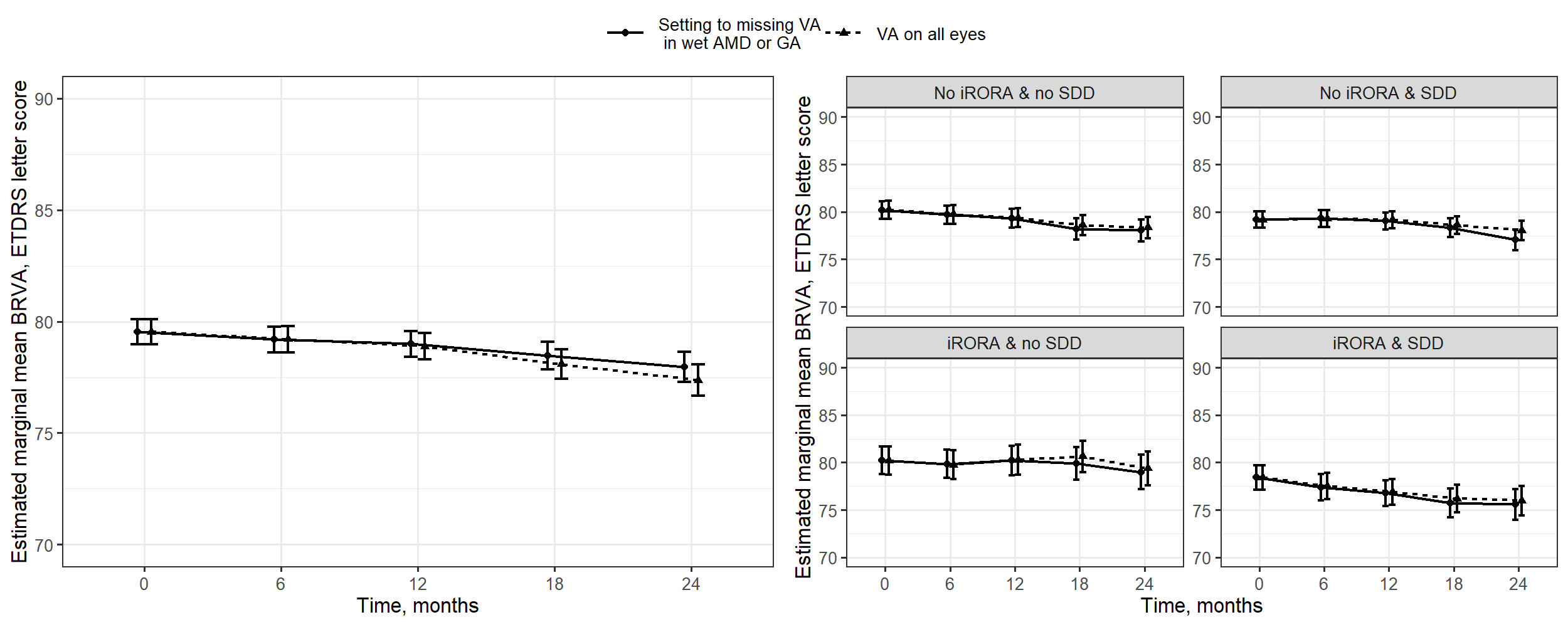

Supplement: Supplementary file 7 — Figure S2. Sensitivity analysis on best recorded visual acuity (BRVA) natural history, setting BRVA to missing in eyes that converted to nAMD or GA using linear mixed effects models (LMEM's)a [file 41433_2025_4062_MOESM7_ESM.tif]
